# Supplementary material for: Stable Configurations of DOXH Interacting with Graphene: Heuristic Algorithm Approach Using NSGA-II and U-NSGA-III
Source: Nanomaterials (Basel). 2022 Nov 21;12(22):4097. doi: 10.3390/nano12224097 (PMC9693072; doi:10.3390/nano12224097)
Supplement: Supplementary file 1 [file nanomaterials-12-04097-s001.zip › nanomaterials-1995157-supplementary.pdf]

Supplementary material: Stable configurations of DOXH interacting with graphene: Heuristic algorithm approach using NSGA-II and U-NSGA-III

Kanes Sumetpipat[1] and Duangkamon Baowan[2]

October 12, 2022

[1]Department of Mathematics and Computer Science, Kamnoetvidya Science Academy, Rayong 21210 Thailand

[2]Department of Mathematics, Faculty of Science, Mahidol University, Rama VI Rd., Bangkok 10400 Thailand

This supplementary document will show more numerical results and graphs for the three systems of (i) interaction between two DOXH molecules, (ii) interaction between a DOXH molecule and a flat graphene sheet and (iii) interaction between two DOXH molecules and a flat graphene sheet.

S-1 Energy behaviors between two DOXHs

The learning curve is plotted in Fig. S1 for Type A1, A2 and A3 of the interaction between two DOXH molecules. It shows that seeds II-5 (Type A1) and seed III-2 (Type A2) have small improvement in the solutions after 200 generations but seed III-3 (Type A3) uses more than 200 generations to converge.

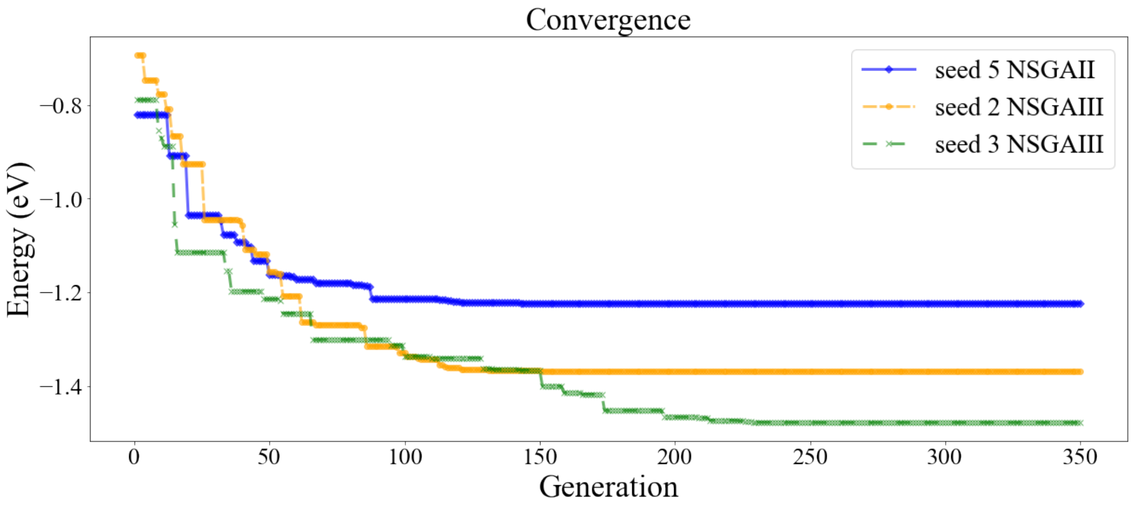

Figure S1: Convergence of algorithms for interacting between two DOXHs.

The three stable configurations between two DOXHs with reference to the Cartesian coordinate system are shown in Fig. S2. Both discrete atomic description and vector representation are depicted.

Moreover to ensure the optimum stage, we plot the energy function in terms of the offset distance between the carbon ring planes of DOXHs and the offset rotational angle on z-axis. The energy profiles for Type A1, Type A2 and Type A3 are plotted in Fig. S3(a), Fig. S3(b) and Fig. S3(c), respectively.

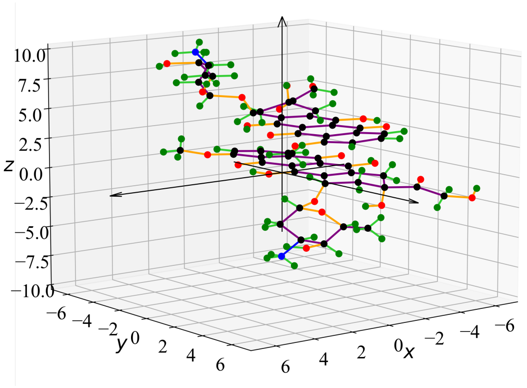

(a) Type A1

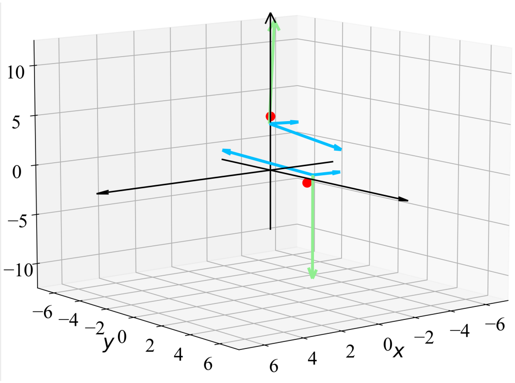

(b) Type A1 (vector)

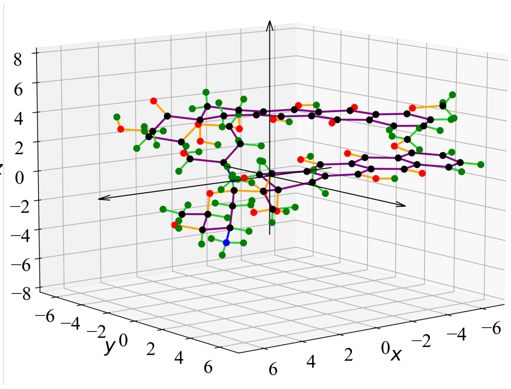

(c) Type A2

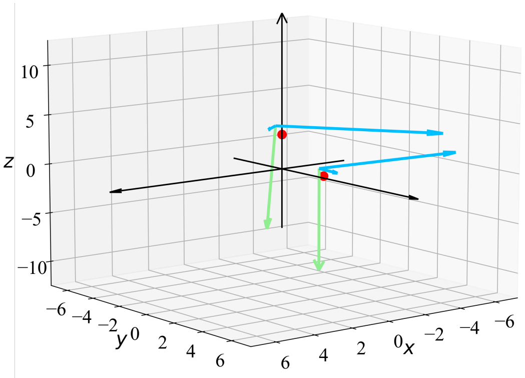

(d) Type A2 (vector)

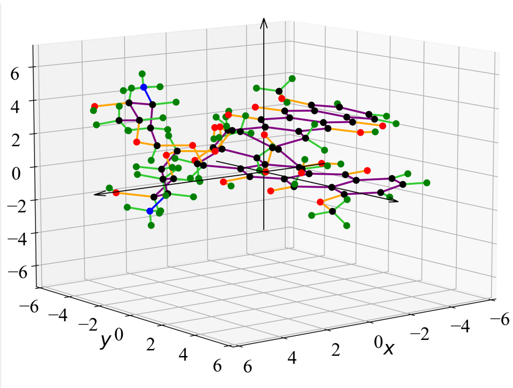

(e) Type A3

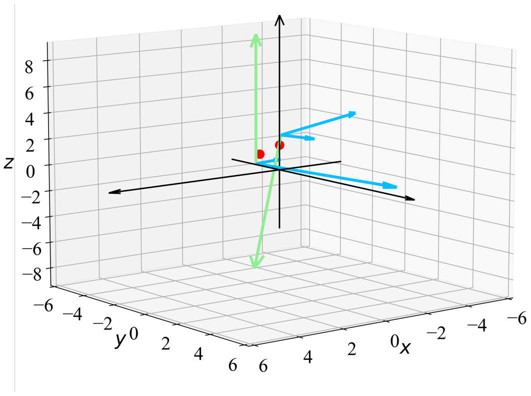

(f) Type A3 (vector)

Figure S2: Atomic structure (left column) and vector representation (right column) for (a),(b) Type A1, (c),(d) Type A2 and (e),(f) Type A3 for three stable configurations of interaction between two DOXHs referencing to Cartesian coordinate system.

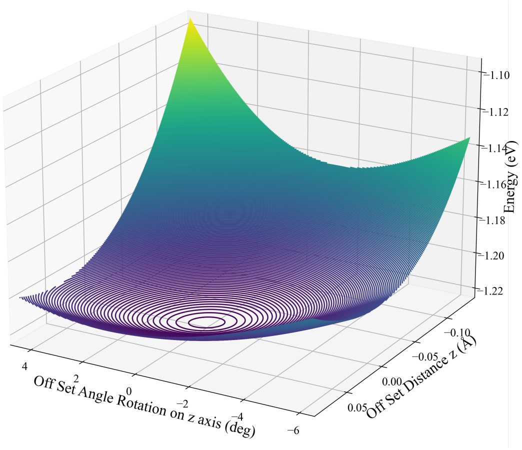

(a) Type A1

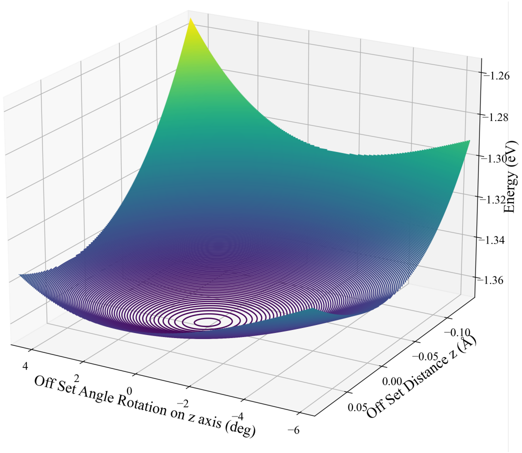

(b) Type A2

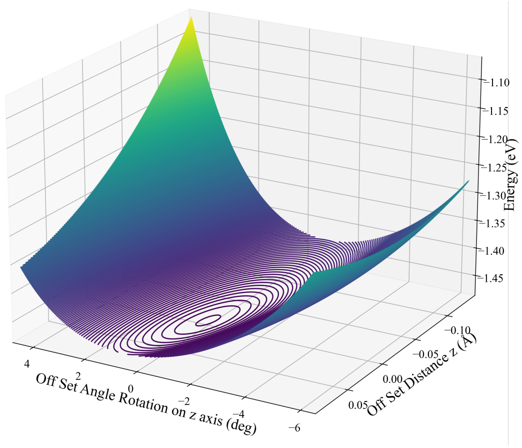

(c) Type A3

Figure S3: Energy level as a function of offset distance between carbon ring planes and offset rotational angle on z-axis for various positions of seed (a) II-5 for Type A1, (b) III-2 for Type A2 and (c) III-3 for Type A3.

S-2 Energy behaviors between DOXH and graphene

We obtain only one stable configuration for the interaction between DOXH and graphene. The optimization learning curve in Fig. S4 is depicted from seed III-15, the algorithm is guaranteed to converge to the stable state after 60 generations.

Both discrete atomic description and the vector representation for the stable configuration is shown in Fig. S5. Further, the three dimensional graphs for the energy level between a DOXH molecule interacting with a flat graphene sheet are illustrated in Fig. S6. For this system, one local minimum is clearly shown which in turn gives only one stable configuration.

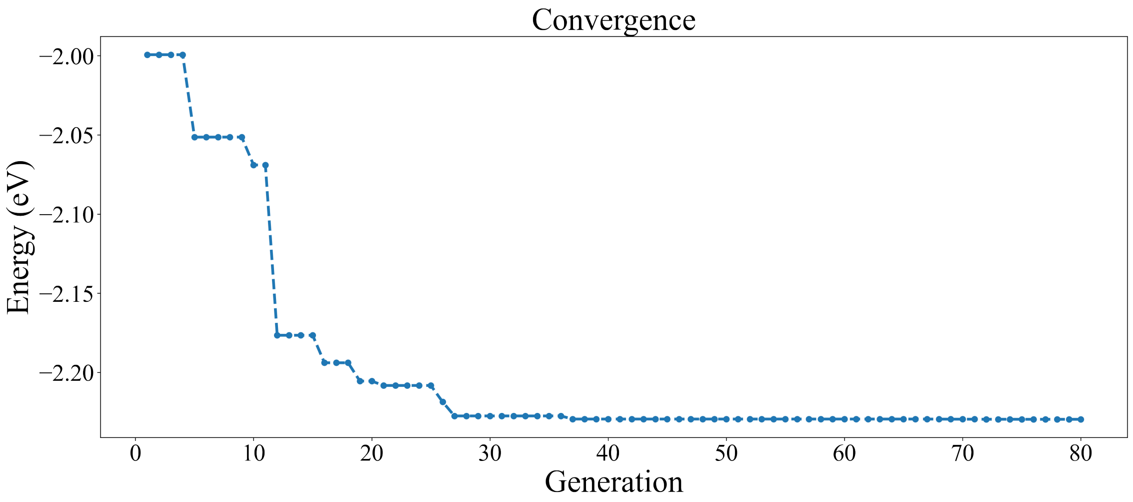

Figure S4: Convergence of algorithm for interaction between DOXH and graphene.

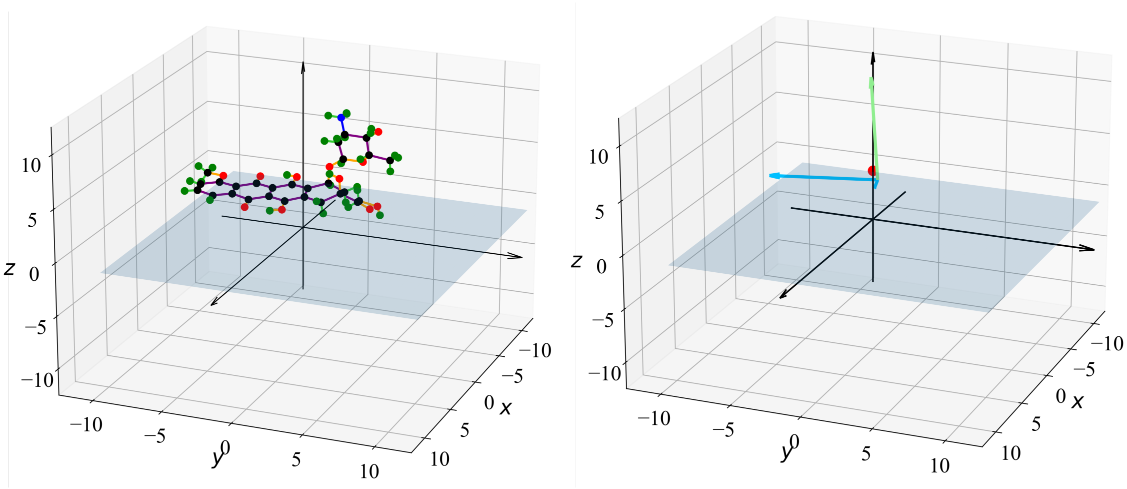

(a) Atomic description (b) Vector representation

Figure S5: (a) Atomic structure and (b) vector representation for stable configurations of interaction between DOXH and graphene sheet referencing to Cartesian coordinate system.

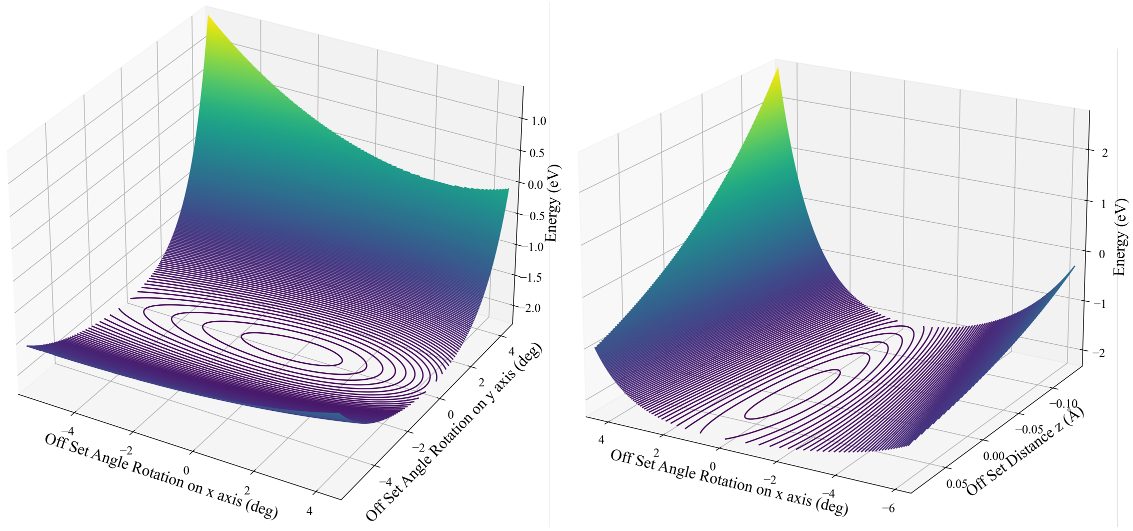

(a) (b)

Figure S6: Energy level as a function of (a) offset rotational angles on x- and y-axis and (b) offset distance z and offset rotational angle on x-axis for various positions for interaction between DOXH and graphene of seed III-7.

S-3 Energy behaviors between two DOXHs and graphene

The optimization learning curves in Fig. S7 belongs to seeds III-9 and III-8 to represent Type B1 and Type B2, respectively. Both results converge to the stable state after 250 generations.

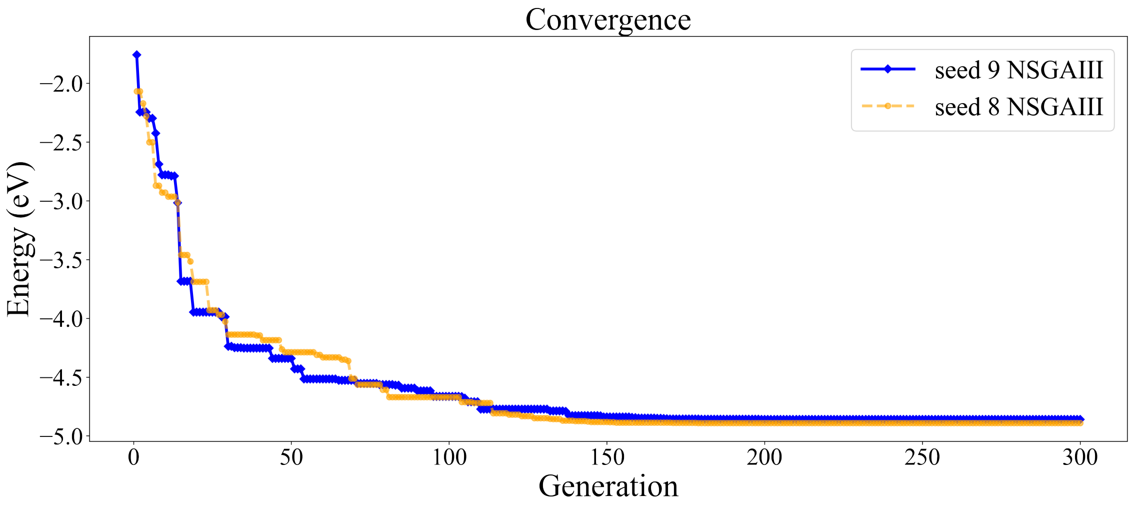

Figure S7: Convergence of algorithms for two DOXHs interacting with graphene.

The atomic structures and their vector representations for the two stable configurations are illustrated in Fig. S8. One local minimum is found for both structures as shown in Fig. S9.

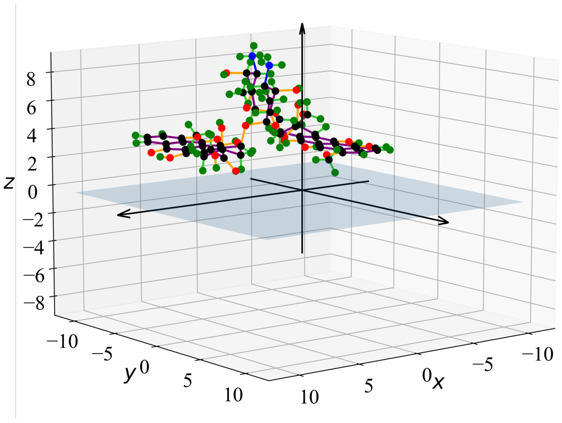

(a) Type B1

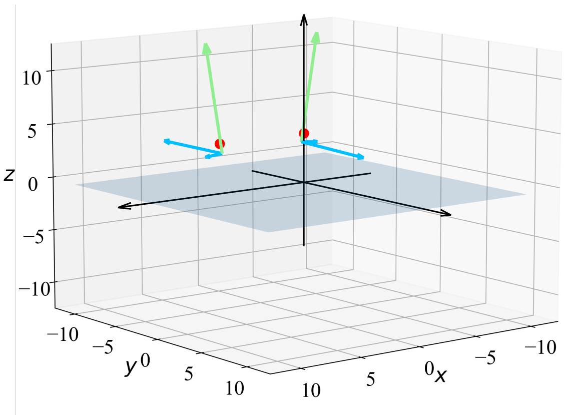

(b) Type B1 (vector)

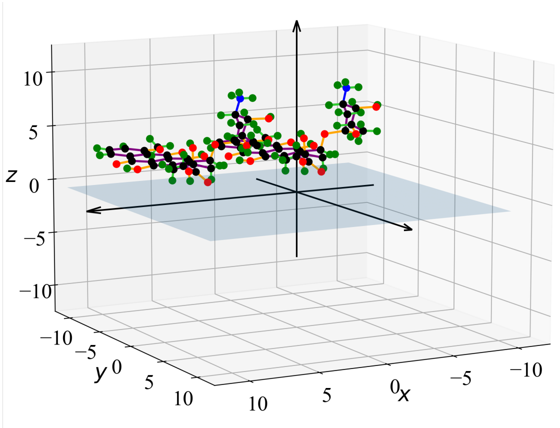

(c) Type B2

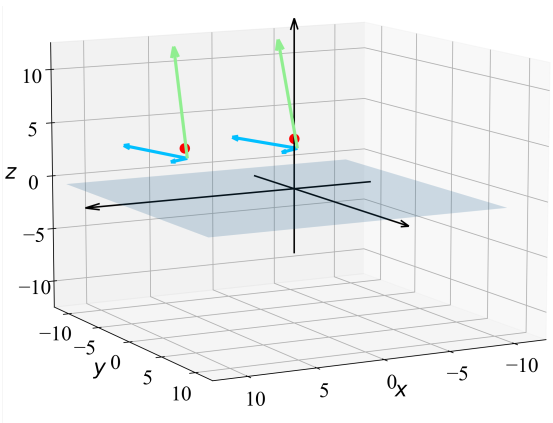

(d) Type B2 (vector)

Figure S8: Atomic structure (left column) and vector representation (right column) for (a),(b) Type B1, and (c),(d) Type B2 for two stable configurations of interaction between two DOXHs and graphene sheet referencing to Cartesian coordinate system.

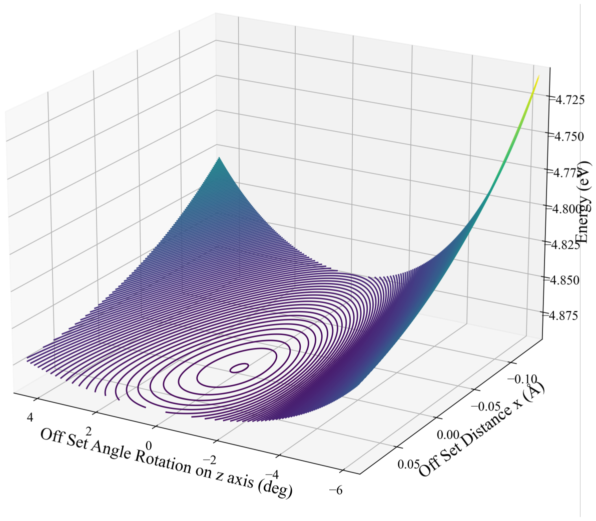

(a) Type B1

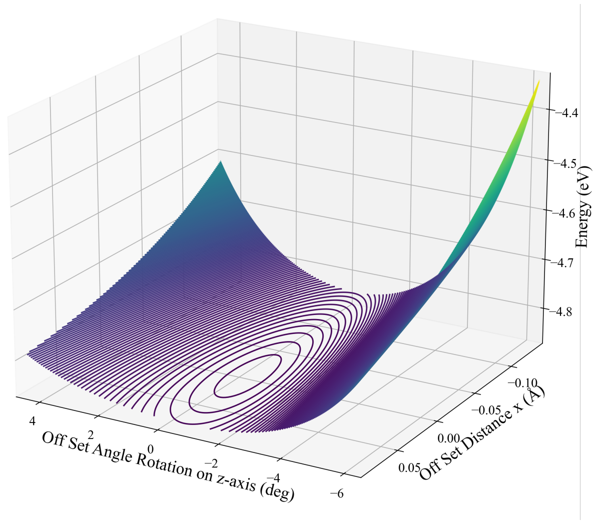

(b) Type B2

Figure S9: Energy level as a function of offset distance between carbon ring planes and offset rotational angle on z-axis for various positions of (a) seed III-9 for Type B1 and (b) seed III-8 for Type B2.
